# Effect of orthokeratology lens wear on axial length elongation in myopic children

**DOI:** 10.3389/fmed.2026.1746705

**Published:** 2026-02-23

**Authors:** Xuejiao Li, Zhijuan Hua, Jie Yin, Liping Xue, Jieying Zhang, Yuan Zhou, Xiaofang Zhang, Xianbo Su, Yingting Zhu, Qin Zhu

**Affiliations:** 1 Department of Pediatric Ophthalmology, The Second People's Hospital of Yunnan Province, Kunming, China; 2 BioTissue/Tissue Tech Inc., Miami, FL, United States

**Keywords:** axial elongation, children, defocus, myopia, orthokeratology lenses

## Abstract

**Purpose:**

To investigate the differences in axial elongation in myopic children wearing orthokeratology lenses (OK lenses) with different back optical zone diameters (BOZD) and single-vision spectacle lenses (SVL).

**Materials and methods:**

This study was a retrospective cohort study that included a total of 230 myopic children aged 8–12 years with spherical equivalent ranging from −5.00 D to −0.50 D, who had been wearing lenses continuously for at least 1 year. The OK lens group consisted of 162 participants, including 86 individuals wearing lenses with a 6.0 mm BOZD (6.0 group) and 76 individuals wearing lenses with a 5.5 mm BOZD (5.5 group). The SVL group included 68 participants. The differences in axial elongation after 12 months of lens wearing were compared among the groups.

**Results:**

After 12 months of lens wear, significant differences were found in axial elongation between the 5.5 group/6.0 group and SVL group (*p* < 0.001). Compared to the SVL group, the median axial elongation was significantly reduced by 78% in the 5.5 group (0.295 vs. 0.065 mm, *p* < 0.001) and by 53% in the 6.0 group (0.295 vs. 0.140 mm, *p* < 0.001). In addition, among patients wearing OK lenses, the axial elongation in the 5.5 group was significantly decreased by 54% compared to the 6.0 group after 12 months of lens wear (*p* < 0.001).

**Conclusion:**

Wearing OK lenses could effectively inhibit axial elongation in myopic children compared to wearing SVL, with better myopia control achieved by using OK lenses with a smaller BOZD.

## Introduction

Myopia is the most prevalent refractive error disease and a leading cause of visual impairment worldwide (1). Its prevalence has been rising steadily, posing a major global public health challenge. Epidemiological studies estimated that the global prevalence of myopia exceeded 28% in 2010 and is projected to affect nearly 50% of the world's population-approximately 5 billion people by 2050 (1, 2). Considerable regional variation exists, with sub-Saharan African countries reporting the lowest prevalence among school-aged children (approximately 3%), whereas certain regions of Southeast Asia report prevalence as high as 80%−90% among high school students (3–5).

China has one of the highest rates of myopia globally, particularly among children and adolescents (6). A national survey in China conducted in 2018 revealed an overall prevalence of 53.6% in individuals aged 6–18 years, including 14.5% among 6-years-olds, 36.0% in primary school students, 71.6% in junior high school students, and 81.0% in senior high school students (7). The prevalence of high myopia is also concerning, reaching 21.9% in high school seniors. In addition, the prevalence is rising rapidly. Compared with December 2019, the overall prevalence in June 2020 increased by 11.7%, with primary school children exhibiting the highest increase of 15.2%. This trend indicates not only an overall increase in myopia prevalence but also a shift toward earlier onset, which substantially raises the risk of high myopia and associated ocular complications, including cataract, glaucoma, macular degeneration, and retinal detachment, potentially resulting in irreversible visual impairment or blindness (8). Collectively, these data underscore the urgent need for effective strategies to prevent and control myopia in children and adolescents.

Early and active intervention to slow down the progression of myopia is of critical importance, as it can reduce the incidence of high myopia and its associated complications, mitigate the impact of pathological myopia on visual function and quality of life, and alleviate the substantial public health and economic burden on society (9, 10). Currently, strategies to control myopia progression can be broadly categorized into pharmacological and optical approaches.

Pharmacological and optical approaches include (1) contact lenses (11), (2) multifocal soft contact lenses (12), (3) peripheral defocusing lenses (13), (4) progressive executive bifocal spectacle lenses (14–16), (5) overnight orthokeratology (OK) (17–19), (6) orthokeratology lenses (20, 21), (7) outdoor exercises (22), (8) pharmacological drugs (23) [also reviewed in Ref. (24, 25)] and (9) their combinations (26–30).

Pharmacological interventions primarily involve the use of atropine eye drops (21, 24, 25, 31), though the exact mechanism by which atropine slows down myopia progression remains unclear. It is hypothesized that atropine acts on receptors in the choroid and sclera, increasing choroidal blood flow, improving scleral hypoxia, and inhibiting scleral remodeling (32). Numerous studies, both domestic and international, have demonstrated that atropine eye drops at various concentrations can effectively slow both refractive error progression and axial elongation. However, side effects such as pupil dilation, photophobia, near vision difficulties, elevated intraocular pressure, and allergic conjunctivitis have also been reported (33). In addition, the optimal concentration and long-term treatment regimen remain undetermined, and some patients experience a rebound effect upon discontinuation of therapy (34).

Optical interventions are primarily based on the principle of peripheral retinal defocus, which has been validated in both animal models and clinical trials (35–37). Among these, orthokeratology (OK) lenses are specially designed as rigid gas-permeable contact lenses with a reverse-geometry design. When worn overnight, these lenses temporarily reshape the cornea, flattening the central cornea, steepening the periphery, thereby creating myopic defocus on the retina. These corneal changes also increase higher-order aberrations (1). An increasing body of evidence indicates that OK lenses are among the most effective methods for myopia control, significantly slowing axial elongation (38, 39), and they have been widely applied for controlling myopia in children and adolescents. However, the efficacy of OK lenses in controlling myopia progression is influenced by multiple factors. In recent years, lenses with smaller optical zone diameters have been explored, aiming to further enhance myopia control efficacy. To evaluate the effect of OK lenses and the differences between optical zone designs, this study retrospectively included school-aged children (8–12 years old) from Yunnan Second People's Hospital who wore either single-vision spectacles (SVL) or OK lenses continuously for 1 year between July 2020 and December 2022. This study aimed to investigate the inhibitory effect of specially designed OK lenses on axial elongation in adolescents, providing scientific evidence to support strategies for controlling myopia progression.

## Materials and methods

### Subjects

This retrospective, non-randomized cohort study included myopic children aged 8–12 years who visited Yunnan Second People's Hospital between July 2020 and December 2022 and met the following inclusion criteria. Participant children were allocated to the different OK lens designs by combinations of the doctors' advice, the parents' thoughts, and the children's choices. Among them, 162 children wore orthokeratology (OK) lenses, comprising 86 children fitted with lenses with a 6.0 mm back optic zone diameter (6.0 mm group) and 76 children fitted with lenses with a 5.5 mm back optic zone diameter (5.5 mm group). An additional 68 children wore single-vision spectacles (SVL). The study was conducted in accordance with the principles of the Declaration of Helsinki and approved by the institutional review board of The Second People's Hospital of Yunnan Province, China.

### Inclusion criteria

The inclusion criteria included (1) age between 8 and 12 years, (2) cycloplegic subjective refraction equivalent spherical refractive error ranging from −5.00 to −0.50 D, with regular corneal astigmatism < 1.50 D, (3) availability of at least 1 year of continuous lens wear and regular follow-up data, (4) no prior history of contact lens wear or other myopia control interventions (including atropine eye drops, defocus soft contact lenses, defocus spectacle lenses, or low-level red light therapy), (5) no ocular or systemic diseases other than refractive error, and no history of ocular surgery, (6) normal binocular visual function before lens fitting, (7) achieved uncorrected visual acuity ≥0.8 after OK lens wear, (8) no significant adverse events observed during OK lens wear.

### Lens specifications

Lens specifications included (1) SVL group: all lenses were of the same brand (Essilor, resin material, refractive index 1.60), to be worn full-time during daytime under normal conditions, with lens replacement performed whenever a change of ≥0.50 D in equivalent spherical refraction was observed; (2) OK lens group: lenses had a two-zone, four-zone reverse-geometry design (Mai'er Kang, Hengtai, Hexafocon-B material) with Dk of 141 × 10^−11^ (cm^2^·mlO_2_)/(s·ml·mmHg) and a central thickness of approximately 0.22 mm. The lenses had diameters ranging from 10.2 to 12.0 mm. All fittings were performed according to manufacturer guidelines and achieved satisfactory lens fit.

### Examinations

All participants underwent a comprehensive ocular examination prior to lens fitting, including (1) refraction, corneal curvature, corneal diameter, and pupil diameter measurement using an automatic refractometer (ARK-1, NIDEK, Japan), combined with cycloplegic subjective refraction using a comprehensive phoropter system (RT-5100/SSC-370/AOS-3000, NIDEK, Japan); (2) corneal topography using a Medmont E300 topographer (Medmont, Australia) to assess corneal shape; (3) axial length (AL) measurement using an IOL-master (AL-scan 500, Zeiss, Germany); (4) Corneal endothelial cell evaluation using a specular microscope (SP-2000P, Topcon, Tokyo, Japan); (5) slit-lamp examination (SLM-7E, Kanghua, China) to exclude ocular conditions that could interfere with OK lens fitting.

### Follow-up schedule

The follow-up schedule included (1) SVL wearers were examined every 3 months; (2) OK lens wearers were examined the day after lens fitting at 1 week, 1 month, and 3 months, and subsequently every 3 months, completing at least 1 year of follow-up. All the eye axis measurements were completed between 9 and 11 in the morning.

### Statistical analysis

Data analysis was performed using EpiData 3.1, and statistical analyses were conducted with IBM SPSS v21.0 (IBM Corp., Armonk, NY, USA). Only the right eye of each participant was included in the analysis. Since not all patients have complete binocular data to avoid the complexity of data statistics caused by inter-eye correlations and to ensure the independence of data points for statistical analysis, this method also conforms to the standard practices in ophthalmic epidemiology. The Kolmogorov-Smirnov test was used to assess normality of continuous variables. Normally distributed variables were expressed as mean ± standard deviation (SD), while non-normally distributed variables were expressed as median (interquartile range). Differences in axial elongation among the three groups (5.5 mm, 6.0 mm, and SVL) were compared using Analysis of Covariance (ANCOVA). The controlled baseline imbalances, baseline age, baseline spherical equivalent refraction (SER), and baseline axial length (AL) were included as covariates in the model. The effect size was reported as partial eta squared ($\eta_{\text{p}}^{2}$). Pairwise comparisons were performed using the Bonferroni adjustment for multiple comparisons. Categorical variables were compared using the chi-square test. For continuous variables, group comparisons were performed using ANOVA for normally distributed data and Kruskal–Wallis *H* tests for abnormally distributed data. A two-sided *p* < 0.05 was considered as statistically significant.

## Results

The 6.0 mm OK lens group had a mean age of 10.49 ± 1.05 years (range 8–12 years), with 51% male participants; the 5.5 mm OK lens group had a mean age of 10.82 ± 1.13 years (range 8–12 years), with 45% male participants; and the SVL group had a mean age of 10.67 ± 1.15 years (range 8–12 years), with 41% male participants. No statistically significant differences were observed in age or sex among the three groups at baseline (*p* > 0.05).

As shown in Table 1, the SVL group had lower SER and shorter AL compared with both the 5.5 and 6.0 mm OK lens groups (both *p* < 0.001). No significant differences in SER or AL were observed between the 5.5 and 6.0 mm OK lens groups at baseline (*p* > 0.05).

**Table 1:** Baseline of different groups.

**Groups**	**5.5 group (*n* = 76)**	**6.0 group (*n* = 86)**	**SVL group (*n* = 68)**
Axial length (mm)	24.68 (24.27–24.99)	24.62 (24.00–25.43)	24.22 (23.88–24.54)
Spherical equivalent (D)	−2.69 (−3.25 to −2.25)	−2.50 (−3.19 to −2.00)	−1.50 (−2.22 to −1.25)
*p*-value (AL)	1.0 (5.5 vs. 6.0)	0.007 (6.0 vs. SVL)	0.004 (5.5 vs. SVL)
*p*-value (SER)	0.08 (5.5 vs. 6.0)	< 0.001 (6.0 vs. SVL)	< 0.001 (5.5 vs. SVL)

Data are expressed as median (Q1-Q3), where Q1 is the 25th percentile and Q3 is the 75th percentile.

As shown in Table 2, significant differences in axial length (AL) elongation were observed among the three groups at both 6 and 12 months (all *p* < 0.001). At 6 months, compared with the SVL group, AL elongation was reduced by 82% in the 5.5 mm OK lens group (*p* < 0.001) and by 70% in the 6.0 mm OK lens group (*p* < 0.001). At 12 months, relative to the SVL group, AL elongation was reduced by 78% in the 5.5 mm group (*p* < 0.001) and by 53% in the 6.0 mm group (*p* < 0.001).

**Table 2:** Changes in axial length at 6 and 12 months in different groups.

**Axial length (mm)**	**5.5 group**	**6.0 group**	**SVL group**
6 months	0.030 (−0.018 to 0.080)	0.050 (0.010–0.100)	0.165 (0.09–0.240)
12 months	0.065 (−0.030 to 0.130)	0.140 (0.043–0.240)	0.295 (0.24–0.438)
*p*-value (6 months)	0.07 (5.5 vs. 6.0)	< 0.001 (6.0 vs. SVL)	< 0.001 (5.5 vs. SVL)
*p*-value (12 months)	< 0.001 (5.5 vs. 6.0)	< 0.001 (6.0 vs. SVL)	< 0.001 (5.5 vs. SVL)

Statistical analysis was performed using the Kruskal–Wallis H test followed by Bonferroni post-hoc multiple comparisons. Data are presented as median (Q1, Q3), where Q1 represents the 25th percentile and Q3 represents the 75th percentile.

**Figure 1:**
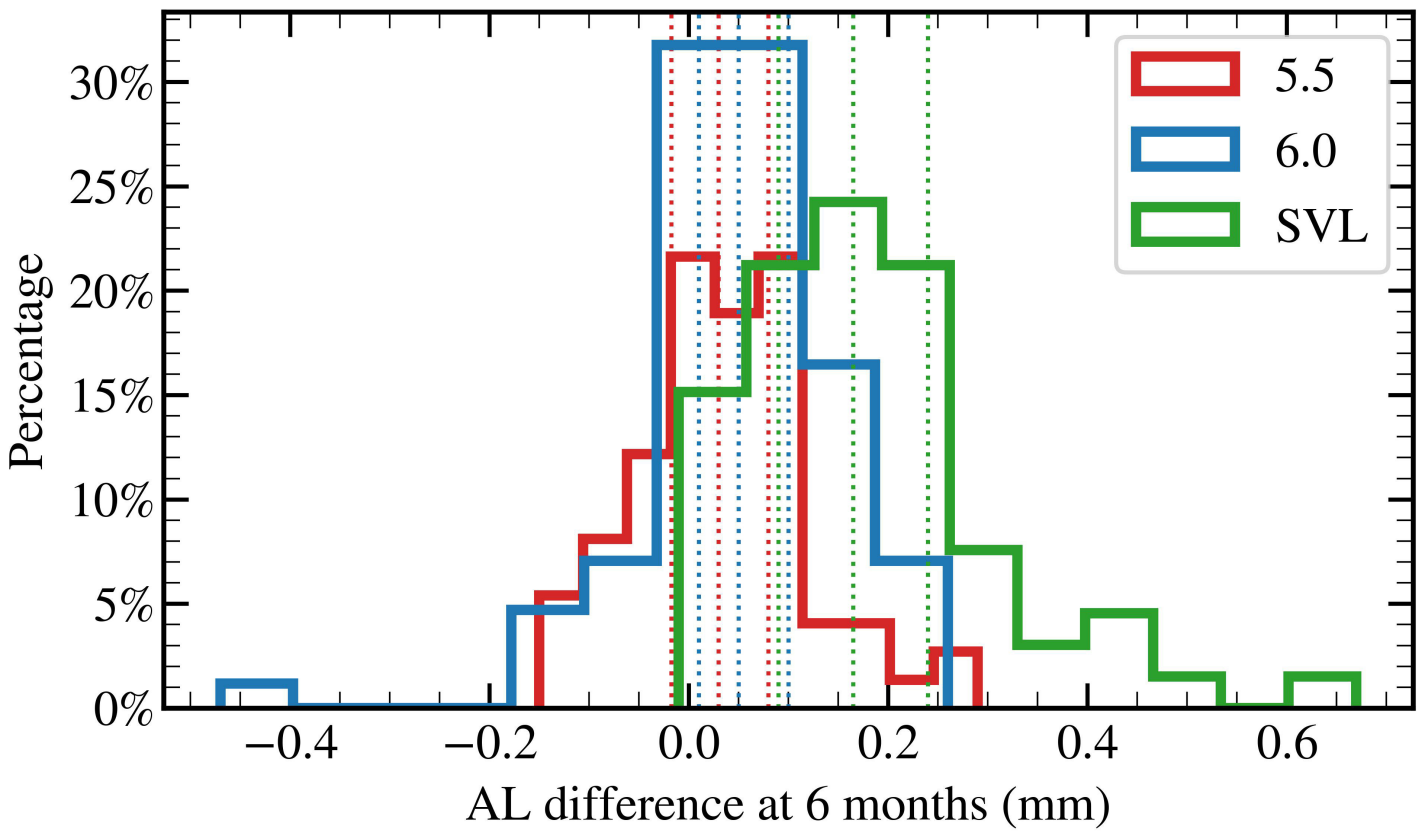
Distribution of axial length changes at 6 months of lens wear.

**Figure 2:**
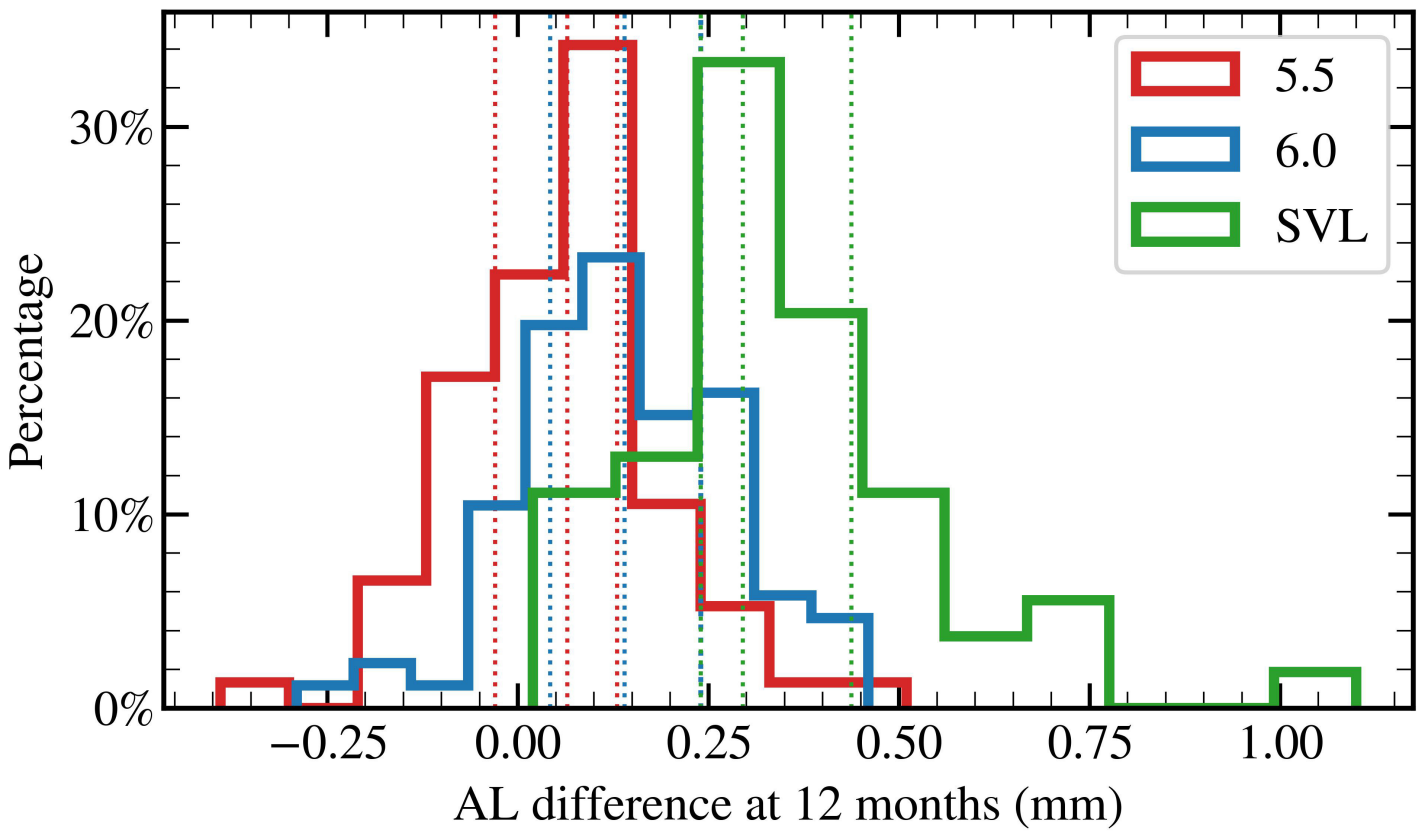
Distribution of axial length changes at 12 months of lens wear.

In addition, at 6 months, AL elongation in the 5.5 mm group was 40% less than that in the 6.0 mm group, although this difference did not reach statistical significance (*p* = 0.06, Figure 1). By 12 months, AL elongation in the 5.5 mm group was 54% less than that in the 6.0 mm group, with a statistically significant difference (*p* < 0.001, Figure 2).

Figure 1 illustrates the distribution of axial length (AL) changes in each group at 6 months of lens wear. Compared with baseline, cases of negative change in the eye axis were observed in the OK lens groups at 6 months (5.5 mm group, 30%; 6.0 mm group, 21%). In contrast, the SVL group exhibited almost no axial shortening at 6 months (3%). For each group, the three dotted lines of the same color correspond to the 25th, the 50th (median), and the 75th percentiles, respectively (also see Table 2).

Figure 2 illustrates the distribution of axial length (AL) changes in each group at 12 months of lens wear. Compared with baseline, negative changes in the eye axis were observed in the OK lens groups at both 12 months (5.5 mm group, 29%; 6.0 mm group, 14%). In contrast, the SVL group exhibited almost no axial shortening at 12 months (1%). For each group, the three dotted lines of the same color correspond to the 25th, the 50th (median), and the 75th percentiles, respectively (also see Table 2).

Given the significant differences in baseline characteristics observed among the groups, an ANCOVA was performed to compare axial elongation at 12 months with adjustment for potential confounders, including age, baseline spherical equivalent refraction (SER), and baseline axial length. The results of the ANCOVA analysis are summarized in Table 3, showing that even after adjusting for these covariates, the difference in axial elongation among the three groups remained highly statistically significant (*F* = 41.515, *p* < 0.001). The effect size was large (partial = 0.285), indicating that the lens design substantially influenced axial growth, independent of baseline ocular parameters. *Post-hoc* pairwise comparisons (see Table 4) revealed that the adjusted axial elongation in the 5.5 mm group was significantly lower than that in the 6.0 mm group (Mean Difference, −0.086 mm, *p* = 0.002) and the SVL group (Mean Difference: −0.279 mm, *p* < 0.001). Similarly, the 6.0 mm group also showed significantly reduced elongation compared to the SVL group (Mean Difference: −0.193 mm, *p* < 0.001).

**Table 3:** ANCOVA analysis.

**Parameters**	***F*-value**	***p*-Value**	**Effect size**
Group (lens type)	41.515	< 0.001	0.285
Age	2.938	0.088	0.014
Baseline SER	0.243	0.622	0.001
Baseline AL	0.011	0.916	< 0.001

Adjusting for these covariates, the difference in axial elongation among the three groups was significant (F = 41.515, *p* < 0.001). The effect size was large (partial = 0.285).

**Table 4:** Adjusted pairwise comparisons.

**Groups**	**Mean difference (mm)**	**Standard error (SE)**	**95% confidence interval**	***p*-Value**
5.5 mm vs. 6.0 mm	−0.086	0.025	−0.145 to −0.027	0.002
5.5 mm vs. SVL	−0.279	0.028	−0.345 to −0.213	< 0.001
6.0 mm vs. SVL	−0.193	0.027	−0.257 to −0.129	< 0.001

The adjusted axial elongation in the 5.5 mm group was significantly lower than that in the 6.0 mm group (Mean Difference, −0.086 mm, *p* = 0.002) and the SVL group (Mean Difference, −0.279 mm, *p* < 0.001). Similarly, the 6.0 mm group also showed significantly reduced elongation compared to the SVL group (Mean Difference, −0.193 mm, *p* < 0.001).

## Discussion

Among the various strategies for myopia control, orthokeratology (OK) lenses are among the earliest and most widely applied interventions, with inhibition of axial elongation serving as the most important indicator of their efficacy. Previous studies have consistently demonstrated that, compared with single-vision spectacles (SVL), OK lens wear can significantly slow axial length (AL) elongation by 46%−81% (40–43). Earlier OK lens studies utilized lenses with a 6.0 mm optic zone diameter. In the present study, after 12 months of wear, the 6.0 mm OK lens group exhibited an AL elongation of 0.140 mm, representing a 53% reduction relative to the SVL group (0.295 mm), which is broadly consistent with previous findings and underscores the superior efficacy of OK lenses in slowing axial elongation.

The precise mechanisms underlying myopia onset and progression remain unclear. Recent clinical and basic research has focused on four main pathways: (1) retinal dopamine release induced by optical defocus to slow myopia development (44, 45), (2) scleral hypoxia and scleral remodeling leading to disproportionate posterior segment elongation (32), (3) alterations in choroidal blood flow affecting ocular growth (46), and (4) peripheral retinal defocus (47). Among these, peripheral retinal defocus serves as the primary mechanistic basis for optical interventions to control myopia progression. Evidence from both animal models and previous human studies indicates that abnormal visual input can influence ocular growth and refractive development (48). Specifically, the peripheral retinal defocus status can modulate axial growth: hyperopic defocus stimulates compensatory axial elongation, promoting myopia progression, whereas myopic defocus can retard axial elongation.

OK lenses reshape the central cornea, temporarily flattening the central curvature while steepening the mid-peripheral cornea, thereby inducing myopic defocus on the peripheral retina and inhibiting axial elongation. In contrast, SVL wear results in central foveal imaging with peripheral hyperopic defocus, whereas OK lens wear provides central foveal imaging with peripheral myopic defocus, which likely explains the superior myopia control efficacy of OK lenses relative to SVL. Based on prior reports, Lenses based on similar defocus principles including peripheral defocus spectacle lenses (49, 50) and defocus soft contact lenses (51) have also been shown to effectively slow myopia progression.

Previous research has suggested that early effects of OK lens wear may be more pronounced. Guo et al. (57) reported that the initial period of wear is associated with more significant axial length control. Consistent with these findings, in the present study, the 6.0 mm group exhibited a 70% reduction in axial elongation relative to SVL at 6 months, compared with 53% at 12 months. Similarly, the 5.5 mm group showed an 82% reduction at 6 months vs. 78% at 12 months, indicating that axial length control may be more prominent during the early phase of OK lens wear. It is worth noting that in this study, the OK lens group showed negative changes in the eye axis at both 6 months (5.5 group, 30%; 6.0 group, 21%) and 12 months (5.5 group, 29%; 6.0 group, 14%) of wearing the lenses. In contrast, the SVL group showed almost no negative changes in the eye axis (6 months, 3%; 12 months, 1%). Although the elongation of the eye axis (AL) is the main mechanism of myopia progression, negative change in the eye axis is also a phenomenon documented in the literature. This is rarely due to scleral anatomical contraction, but more often due to transient choroidal thickening (52, 53), re-distribution of corneal tissue after wearing corneal reshaping lenses (54), circadian rhythm of eye axis length (55), the state of the ciliary muscle (accommodation) (56), or measurement errors.

Furthermore, prior studies have demonstrated that the optic zone diameter influences OK lens efficacy. Guo et al. (57) reported in 2021 that AL elongation over 1 year was significantly lower in the 5 mm optic zone group compared with the 6 mm group (0.04 ± 0.15 mm vs. 0.17 ± 0.13 mm, *p* = 0.001). Zhang et al. (58) found that AL elongation in the 5 mm optic zone group (0.09 ± 0.14 mm/year) was lower than in the VST design 6.2 mm group (0.26 ± 0.14 mm, *p* = 0.002) and the CRT design 6.0 mm group (0.32 ± 0.18 mm, *p* < 0.0001). In the present study, after 12 months, the 5.5 mm group exhibited 54% slower AL elongation compared with the 6.0 mm group (*p* < 0.001), consistent with previous reports and indicating that lenses with smaller optic zones may more effectively slow axial growth. Multiple factors can influence OK lens efficacy, including pupil diameter (59), initial age of wear (36, 60), baseline refractive error (60), higher-order aberrations (61), amount of defocus (62), position of the defocus ring (63), and asphericity of the treatment zone (64). Although this study did not conduct any relevant measurements by further optimizing the lens design through measures such as reducing the diameter of the optical zone, it is possible to reduce the defocus ring, increase higher-order aberrations and defocus amount, making the shape of the plastic zone more non-spherical. This also explains the effect of increasing the inhibitory effect of OK lenses on eye axis elongation. It might also be a possible cause for the better efficacy of myopia control in the small optical zone group in this study. By employing lenses with smaller optic zones, the defocus ring can be reduced, higher-order aberrations and defocus can be increased, and the treatment zone can be more aspheric, thereby enhancing inhibition of axial elongation. This may explain the superior myopia control observed in the 5.5 mm group in the present study. Small optic zone lenses are increasingly adopted to improve OK lens efficacy, particularly in patients with smaller pupils. Prior studies have suggested that positioning the defocus ring within the pupillary margin enhances axial control (39), whereas conventional 6.0 mm lenses might be less effective in such patients.

This study has several limitations.

Firstly, the limitation is the possible selection bias due to a non-randomized retrospective study. As a result, there would be a certain degree of bias in the selection of the patients' wearing methods, which might affect the correlation between OK lenses and the growth of the eye axis. A prospective randomized cohort study might further verify the correlation between OK lenses and the inhibition of eye axis growth. Secondly, only including data from the right eye for analysis would reduce the relevance of the statistical results. In future studies, a mixed-effects model could be used to incorporate data from both eyes, allowing for better handling of inter-eye dependencies. Thirdly, at baseline, the SVL group had significantly lower SER and AL compared with the OK lens groups. The main reason for the baseline difference might be selection bias. Given the significant differences in baseline characteristics observed among the groups, an ANCOVA was performed to compare axial elongation at 12 months while adjusting for potential confounders, including age, baseline spherical equivalent refraction (SER), and baseline axial length. The results showed that the differences in eye axis elongation among the three groups still had a highly significant statistical difference. Given the absence of significant differences in age and sex at baseline, factors influencing myopia progression were primarily baseline refractive error and AL. Higher baseline refractive error and longer AL might be associated with faster progression (65). Therefore, without intervention, the OK lens groups would theoretically have been at higher risk of myopia progression, suggesting that the observed efficacy might be underestimated. The next step requires the design of a prospective randomized clinical trial to further prove the effectiveness of this method in inhibiting the elongation of the eye axis and to identify its influencing factors. Fourthly, there might be a lack of research on visual quality and safety. A smaller optical zone might increase the risk of glare. In subsequent studies, standardized visual quality assessment should be conducted to further balance the effect of myopia control with visual quality. At the same time, since wearing orthokeratology lenses poses risks of corneal hypoxia and mechanical damage, this study might require safety assessment for wearing orthokeratology lenses. Therefore, safety assessment standards need to be established to further confirm the safety of wearing orthokeratology lenses. Moreover, this study was conducted in a single center only, lacking comparative data from multiple centers. Finally, this study only used a single brand of OK lenses, and there was no comparative study with other brands. In the next step, multiple centers might be combined to collect more samples and OK lenses from multiple brands. This would reduce the interference of confounding factors and enhance the credibility of the results.

Despite these limitations, given the recent introduction of small optic zone OK lenses and limited existing data, the present study provides additional evidence regarding the effect of different optic zone designs of the same brand on AL elongation. Overall, our findings indicate that OK lenses are more effective than SVL in controlling myopia progression, with the 5.5 mm small optic zone lenses exhibiting superior efficacy, slowing down myopia progression by 78% at 12 months relative to SVL. These results provide a scientific basis for selecting appropriate myopia control strategies in school-aged children.

**Figure 3:**
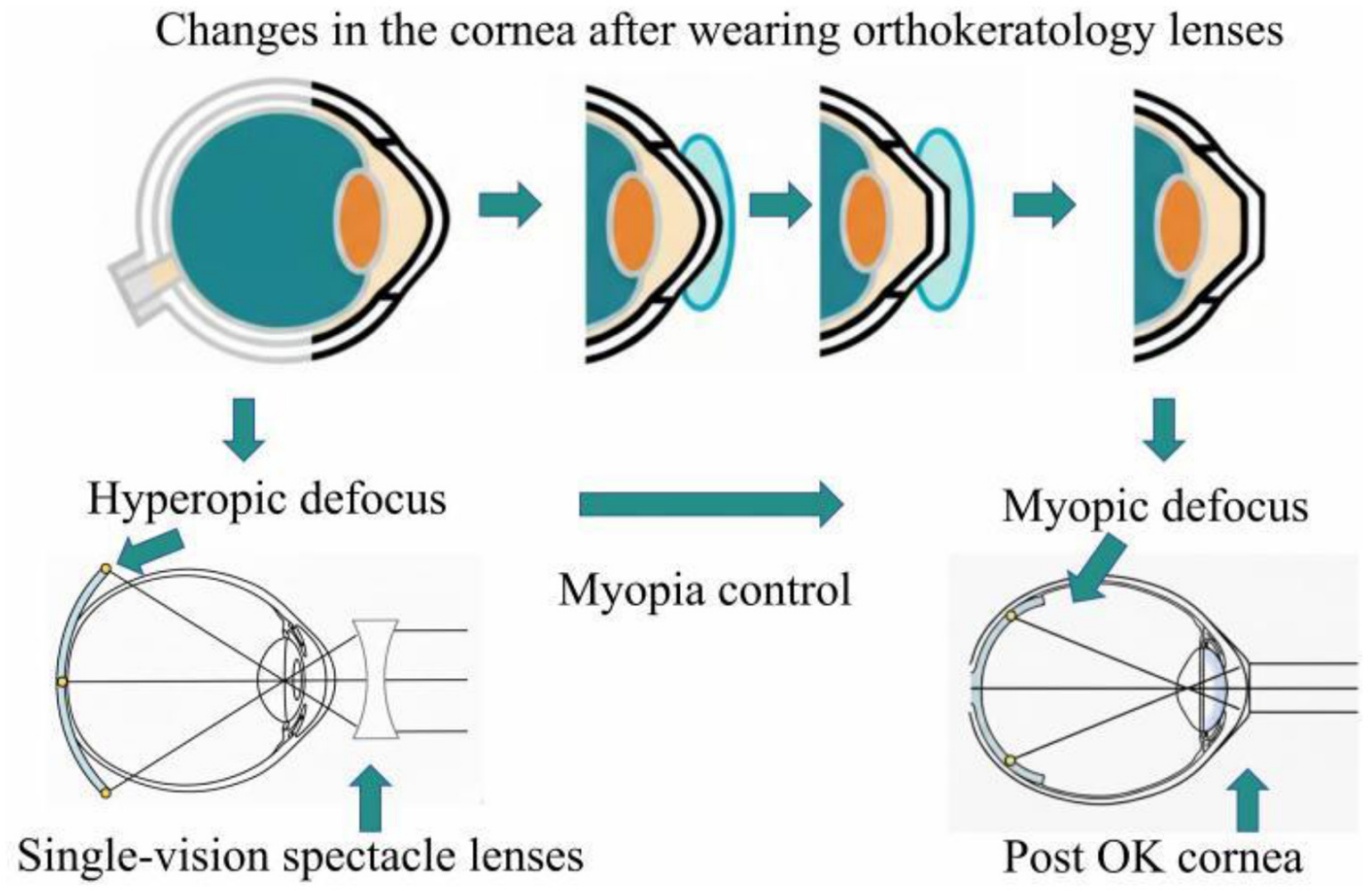
Diagram of the mechanism by which OK lenses control myopia.

As summarized in Figure 3, which illustrates the changes in corneal morphology after wearing orthokeratology lenses. Wearing single-focus glasses results in far sighted defocus at the periphery of the retina while wearing OK lenses result in myopic defocus at the periphery of the retina. Hyperopic defocus is transformed into myopic defocus to achieve OK lenses' control of myopia.

## Data Availability

The original contributions presented in the study are included in the article/supplementary material, further inquiries can be directed to the corresponding authors.
